# A Retrospective Analysis of the Clinical Impact of 939 Chest Radiographs Using the Medical Records

**DOI:** 10.1155/2012/862198

**Published:** 2012-12-20

**Authors:** Mats Geijer, Liz Ivarsson, Jan H. Göthlin

**Affiliations:** ^1^Center for Medical Imaging and Physiology, Lund University and Skåne University Hospital, 221 85 Lund, Sweden; ^2^Department of Radiology, Sahlgrenska University Hospital, 413 45 Gothenburg, Sweden

## Abstract

*Objective*. Between one-third and half of all radiology examinations worldwide are probably chest studies. The aim of the current study was to retrospectively evaluate the clinical influence of chest radiography. *Methods*. In a tertiary referral hospital, 939 consecutive daytime chest radiography examinations were evaluated. The outcome was classified as normal, incidental, or pathologic. The referring physician's reaction to radiologic outcome was classified as highly expected, moderately expected, or unexpected. The influence on the patients' treatment was divided into four groups from major to no influence. *Results*. In all, 71.6% of the studies had a highly expected outcome. Moderately expected or unexpected outcomes were noted in 36.6% of 500 pathologic examinations. Unexpected outcome was noted in 11.6% of all studies. The radiologic outcome influenced treatment in 65.4% of patients where pathology was demonstrated. Patients with normal or incidental findings had treatment influenced in 1/3 of the cases. Unexpected findings influenced treatment more than moderately expected findings. When radiological findings were highly expected, treatment was influenced in less than half of the cases. Surprisingly few chest radiology examinations were commented upon in the medical records.

## 1. Introduction

According to an old World Health Organization (WHO) survey (cited in [1]), about half of all radiology examinations worldwide are of chest, and it is the most frequently performed radiologic study in US hospitals [2]. In our hospital, around 30% of all examinations in the radiology department are chest radiographs according to annual audits, and despite the low cost per examination, thus consume a considerable part of the department resources. Chest radiography is a technically easily performed examination and fairly easy to analyze considering its clinical impact. 

The real value of radiology for the referring physician and the patient can be assessed by analyzing its clinical utility. One obvious way of doing this is to register and analyze how and when radiology has induced treatment changes or been used to monitor treatment. A large number of papers report the lack of clinical utility or efficacy of routine admission [2], screening [3], and preoperative [4, 5] and postoperative chest X-rays [6]. It has been reported that routine admission chest radiography has the highest utility in patients who actually have a clinical indication for chest radiography [7] and that chest radiography demonstrating pathology has a higher influence on clinical treatment decisions than chest radiography without pathologic findings [7–9]. Perusal of the literature has not revealed any article on the true influence of chest radiology on diagnosis, treatment, and monitoring of disease from a clinical point of view except two papers on the clinical utility of chest X-rays in general practice [8, 9]. No studies on the clinical utility of chest X-rays in a hospital setting have been found. Nor has a perusal of the literature revealed any report neither on how frequently radiologic outcome is referred to in clinical records, nor if they are really noted.

The aim of the current study has been to retrospectively evaluate the clinical influence of chest radiography in a large number of examinations by (1) assessing the relationship between the radiologic outcome and the clinical response, (2) assessing the relationship between outcome and influence on clinical treatment, and (3) assessing to which extent the radiologic outcome was noted and referred to in the medical records. 

## 2. Materials and Methods 

One thousand consecutive office-hour chest radiographs requested from seven large clinical departments performed on 588 male and 412 female patients were evaluated. The age range was 17–98 years (median 66 years). The age range for male patients was 17–91 years (median 65 years), and the age range for female patients 18–98 years (median 69 years). After exclusion of 61 patients with incomplete medical records, 939 cases remained to evaluate.

The study setting was a tertiary referral hospital in which about thirty percent of all radiological examinations are of the chest according to yearly audits. All referrals were listed at the end of the day. The examinations were performed during a six-week period. 

The outcome of chest radiography was classified as normal, incidental, or pathologic. Normal was defined as without incidental or pathologic findings in the parenchyma, pleurae, or hila. Incidental findings were defined as a chest examination showing findings deviating from normal but without need for medical treatment. Incidental findings included changes such as aortic calcifications, elongated thoracic aorta, minor pleural calcifications or scars, or mild chronic obstructive disease. Pathologic findings were those in need of medical treatment, such as pneumonic infiltrates, cardiac incompensation, pneumothorax, or rib fractures. At the time of the study electronic medical records had not been fully implemented, and medical records were available on paper and on microfilm. The referring physician's reaction to the radiologic outcome (how the referring physician evaluated the report) was divided into three groups (highly expected results, moderately expected results, and unexpected results). Highly expected results were those where the clinician received confirmation of a clinical suspicion of pathology such as pneumonia or a normal radiography report on a routine study done for screening purposes. Moderately expected results were those where clinical suspicion was not very high but was confirmed, or another chest pathology than the suspicion given in the referral form was present to account for symptoms. Unexpected results were those where the radiologic findings were contrary to the clinical suspicion, such as normal chest radiography on a patient with clinical suspicion of pneumonia. The influence of the chest radiography examination on the patients' treatment was divided into four groups: major influence, moderate influence, minor influence, and no influence. Major influence represented a radiology report that initiated or changed medical treatment. Moderate influence represented cases where the outcome of chest radiography confirmed the tentative clinical diagnosis, and treatment was started. Minor influence represented cases where radiology confirmed already diagnosed disease and induced no change in treatment. No influence represented cases where radiology did not influence treatment. All available medical records including daily notes, nurses' records, summaries, and the request forms for chest radiography were analyzed. Primarily it was noted whether the medical records contained any written reference to the radiological examination, apart from the proper radiology report.

Statistical analysis was performed using StatView for Windows 4.57 (Abacus Concepts, Inc.). Descriptive statistics are presented as median and range. The significance of the results was calculated using Pearson's chi-squared test, where a *P* value < 0.01 was considered statistically significant. 

## 3. Results

Most requests for radiography (92.9%) came from the departments of internal medicine (*n* = 260), general surgery (*n* = 203), thoracic surgery (*n* = 184), cardiology (*n* = 125), and intensive care (*n* = 100). There were 776 examinations (82.6%) performed on inpatients and 163 on outpatients. Clinical indications accounted for 778 cases (82.9%). Routine preoperative examinations accounted for 126 cases, and 31 examinations were performed routinely before coronary angiography.

Radiologic outcome was pathologic in 500 cases (53.2%), showed incidental findings in 77 (8.2%), and was normal in 362 cases (38.6%). Regardless of radiologic findings, 71.6% of all studies had a clinically highly expected outcome (Table 1). Unexpected outcome was noted in 11.6% of all studies. Moderately expected or unexpected outcomes were noted in 36.6% of the 500 pathologic examinations. The results in Table 1 are highly significant (*P* < 000.1), where the largest deviation from expected based on the marginals of the table was among unexpected normal and unexpected pathologic findings.

Chest radiography had a major influence on treatment in 491 cases (52.3%), a moderate influence in 23.0%, a minor influence in 17.7%, and no influence in 7.0% (Table 2). It had a major influence on treatment in 65.4% (327/500 patients) when pathology was demonstrated (Table 2). In the groups of normal and incidental findings, there was a major influence on treatment in about 1/3 of the cases. The results in Table 2 are highly significant (*P* < 000.1), where the largest deviation from expected based on the marginals of the table are fewer cases than expected of pathologic findings with minor or no influence on treatment.

The radiographic outcome was highly expected in 672 cases (71.6%), moderately expected in 158 (16.8%), and unexpected in 109 cases (11.6%). Unexpected findings had a major influence on treatment in 76.1% (83 of 109 cases), somewhat more than that for moderately expected findings (Table 3). When the radiological findings were highly expected, the choice of treatment was altered or influenced in less than half of the cases. The results in Table 3 are highly significant (*P* < 000.1), where mainly the unexpected findings were not distributed according to the marginals of the table. There were more unexpected cases with major influence on treatment and fewer cases with minor or no influence on treatment than expected.

More than half of the radiological examinations were not referred to in the clinical records. Several were not even noticed. The lowest rate was noted for routine preoperative chest radiographs and radiography prior to coronary angiography. The highest annotation rate of the radiologic outcome in the clinical medical records, 58.7%, occurred when the radiologic outcome had a major influence on treatment (Table 4). Successively lower annotation rates were noted for the groups of medium and minor influence. 

Preoperative examinations or examinations performed before coronary angiography were studied separately. Their clinical influence was low. Totally 17.8% of the 157 examinations were judged to have had a major influence on treatment, 24.2% a medium influence, 38.2% a minor influence, and 19.7% no influence. Also the rate of annotation in the medical records was low. The results from preoperative chest examinations were noted in the medical records in 8.7% (11/126) and examinations before coronary angiography in 12.9% (4/31). 

## 4. Discussion

The main purpose of the current study was to evaluate the influence of daytime chest radiography on the clinical treatment of patients by a retrospective analysis of medical records but also to evaluate how the radiology reports were handled. The value of chest radiography in symptomatic emergency patients such as those encountered at night and during weekends is well known and not the subject of the current study. It might be argued that a retrospective study which is based on medical files and radiology reports would have less value than a prospective study. However, it has been shown that case notes do contain sufficient information to evaluate clinical performance retrospectively [10].

In the current study, moderately expected and unexpected outcomes were noted in 36.6% of the 500 pathologic examinations. Unexpected outcome was noted in 11.6% of all examinations. Chest radiographs demonstrating pathology had a higher rate of influence on the clinical treatment than radiographs demonstrating incidental or normal findings. This is consistent with the findings from other reports on hospital populations [7] or patients referred by general practitioners [8, 9].

The clinicians' reactions to the outcome of the radiologic examination was judged based on the notes in the medical records and also on our own clinical experience as the medical records sometimes were incomplete. In a high proportion of cases, we were unable to find any reference to the outcome of radiology in the medical records apart from the radiology report itself. In those cases we judged the clinical interest in the radiology examination to be very low and the examinations to a very large extent being routine without any clinical relevance like preoperative and pre coronary angiography examinations. The examinations which were most unexpected, and also influenced treatment most, were those with pathologic findings, and in those cases there was also a higher rate of annotation of the radiologic outcome in the medical records.

The influence on clinical treatment was judged to be high if there were medical notes about the radiologic outcome and about the consequences of the outcome. However, most medical records were not that eloquent, and in many cases we had to infer changes in treatment from changes in medication in the case notes, abstaining from planned operations, and so forth.

It was surprising that so many radiologic examinations went by unnoticed or without annotation. Totally, in more than half of the cases, there was no annotation in the medical records about the outcome of the study. The examinations where the outcome was pathologic or had an influence on the clinical treatment had a higher rate of annotation. Routine tests without influence on medical treatment should preferably be avoided, since they only take up valuable resources and disperse the information obtained from other tests for clinical reasons, an argumentation which is valid for all routine tests [11, 12].

Routine preoperative examinations and examinations performed before coronary angiography had a very low rate of influence on treatment, even lower than that of the entire group of examinations with highly expected results, corresponding to results in previous studies on preoperative examinations [11]. There was also a very low rate of annotation of the outcomes of preoperative radiography in the medical records in the current study. In a review on preoperative procedures before abdominal surgery, chest radiography was recommended for high-risk patients only [13]. It has little value in selecting patients who are at risk for perioperative complications [14]. In a meta-analysis of studies performed on European and North American patient populations, it was concluded that routine preoperative chest radiography was superfluous [5]. Likewise, chest radiography before coronary catheterization has proved to have very little clinical value, causing none of 240 coronary arteriograms to be postponed or cancelled in one study [15] and an influence on the procedure in only 12 of 340 arteriograms in another study [16]. In that study, chest radiography before coronary angiography was significantly more helpful in congenital heart disease and dilated cardiomyopathy than in ischemic heart disease [16].

It was, of course, impossible to exactly assess the clinical physicians' rate of expectation of what radiology would yield. It was also difficult to assess the extent to which the radiology reports influenced diagnosis and treatment in the current study, since the evaluations have been made retrospectively on the data originally provided by the referring clinicians. It seems reasonable to suppose that some degree of misjudgment has been made, and we may have overestimated the clinical influence of the examinations but the main conclusions are probably valid. 

A special problem has been the assessment of the influence of reports with no pathology. The value of the negative examination should, however, not be underestimated [17], although, to our notice, no studies on that subject have been made. On the other hand, in a population with low prevalence of disease and many normal findings, there may be an increased number of false positive findings [18, 19]. As discussed by Kundel [18], disease prevalence has a high impact on the positive predictive value of a test. In an example presented in that paper, it is shown that as the prevalence of disease is changed, the positive predictive value of a diagnostic test is also changed. For instance, if the prevalence of disease in one population is 5% but 0.05% in another, a diagnostic test with a sensitivity of 95% and a specificity of 99% would have a positive predictive value of 83% in the population with a disease prevalence of 5%, but only 4.5% in the population with a disease prevalence of 0.05% [18]. Kundel goes on to discuss how this fact may influence reader performance in radiologic studies, where in patient populations with low disease prevalence readers may unconsciously adjust their attitudes to reduce the number of false positives, which will result in a reduction also in the number of true positives, exemplified by a number of screening studies on lung cancer and pulmonary tuberculosis [18]. It would be reasonable to assume that disease prevalence also affects daily clinical radiologic practice in a similar manner.

Examinations on patients without chest symptoms, such as preoperative examinations, examinations before coronary angiography, routine controls or followup, or purely administrative routine chest radiology had a very low rate of pathologic findings and thus in most cases had a highly expected outcome. They had a low clinical impact and should probably have been avoided. Also routine admission radiography may fall into this category [2], as well as routine chest radiography in the intensive care unit (ICU). In a report on a change of strategy in an ICU, from routine to on-demand chest radiography, the same amount of abnormalities was detected on a reduced number of chest radiographs without affecting the readmission rate, ICU, or hospital mortality rates [20]. In a study by Malnick et al., chest radiography had significant impact on patient management only when there were relevant findings on physical examination or a clear clinical indication for performing the test [7].

In conclusion, there was a low rate of annotation about the chest radiology examinations in the medical records. Many chest radiology reports did influence decision making regarding diagnosis and treatment. The clinical utility of chest radiography thus appears fairly good, especially considering that the examination is rather inexpensive. The clinical utility is highest in patients with clinical symptoms and less in purely routine examinations on patients without symptoms. 

## Figures and Tables

**Table 1:** Concordance between radiographic outcome and clinician's expectations in 939 chest radiographs, grouped according to the chest radiography findings. Normal studies had a higher degree of expected outcome than pathologic studies.

Result	Highly expected	Moderately expected	Unexpected	Total
Normal	291 (80.4%)	64 (17.7%)	7 (1.9%)	362 (100.0%)
Incidental	64 (83.1%)	8 (10.4%)	5 (6.5%)	77 (100.0%)
Pathologic	317 (63.4%)	86 (17.2%)	97 (19.4%)	500 (100.0%)

Total	672 (71.6%)	158 (16.8%)	109 (11.6%)	939 (100.0%)

**Table 2:** Alteration or influence on treatment by 939 radiographic chest examinations, grouped according to chest radiographic outcome. Pathologic studies had the highest rate of influence on treatment choices.

	Major	Moderate	Minor	No influence	Total
Normal	134 (37.0%)	78 (21.5%)	99 (27.3%)	51 (14.1%)	362 (100.0%)
Incidental	30 (39.0%)	24 (31.2%)	18 (23.4%)	5 (6.5%)	77 (100.0%)
Pathologic	327 (65.4%)	114 (22.8%)	49 (9.8%)	10 (2.0%)	500 (100.0%)

Total	491 (52.3%)	216 (23.0%)	166 (17.7%)	66 (7.0%)	939 (100.0%)

**Table 3:** Alteration or influence on treatment by 939 chest radiography examinations, grouped according to the referring physicians' anticipation of the chest radiography outcome. Unexpected chest radiography results influenced treatment to a higher degree than moderately or highly expected results.

	Major	Moderate	Minor	No influence	Total
Highly expected	302 (44.9%)	171 (25.4%)	140 (20.8%)	59 (8.8%)	672 (100.0%)
Moderately expected	106 (67.1%)	30 (19.0%)	17 (10.8%)	5 (3.2%)	158 (100.0%)
Unexpected	83 (76.1%)	15 (13.8%)	9 (8.3%)	2 (1.8%)	109 (100.0%)

Total	491 (52.3%)	216 (23.0%)	166 (17.7%)	66 (7.0%)	939 (100.0%)

**Table 4:** Rate of annotations in the medical records about the outcome of chest radiography of 939 examinations, grouped according to influence of the chest radiography outcome on treatment. Cases with higher influence on treatment were to a higher degree remarked on in the medical records.

	Annotation	No annotation	Total
Major	288 (58.7%)	203 (41.3%)	491 (100.0%)
Moderate	102 (47.2%)	114 (52.8%)	216 (100.0%)
Minor	23 (13.9%)	143 (86.1%)	166 (100.0%)
No influence	11 (16.7%)	55 (83.3%)	66 (100%)

Total	424 (45.2%)	515 (54.8%)	939 (100.0%)

## References

[1] Tape TG, Mushlin AI (1986). The utility of routine chest radiographs. *Annals of Internal Medicine*.

[2] Verma V, Vasudevan V, Jinnur P (2011). The utility of routine admission chest X-ray films on patient care. *European Journal of Internal Medicine*.

[3] Kubik A, Parkin DM, Khlat M, Erban J, Polak J, Adamec M (1990). Lack of benefit from semi-annual screening for cancer of the lung: follow-up report of a randomized controlled trial on a population of high-risk males in Czechoslovakia. *International Journal of Cancer*.

[4] Gagner M, Chiasson A (1990). Preoperative chest x-ray films in elective surgery: a valid screening tool. *Canadian Journal of Surgery*.

[5] Archer C, Levy AR, McGregor M (1993). Value of routine preoperative chest x-rays: a meta-analysis. *Canadian Journal of Anaesthesia*.

[6] Munro J, Booth A, Nicholl J (1997). Routine preoperative testing: a systematic review of the evidence. *Health Technology Assessment*.

[7] Malnick S, Duek G, Beilinson N (2010). Routine chest X-ray on hospital admission: does it contribute to diagnosis or treatment?. *Israel Medical Association Journal*.

[8] Geitung JT, Skjærstad LM, Göthlin JH (1999). Clinical utility of chest roentgenograms. *European Radiology*.

[9] Speets AM, van der Graaf Y, Hoes AW (2006). Chest radiography in general practice: indications, diagnostic yield and consequences for patient management. *British Journal of General Practice*.

[10] Charny MC, Roberts GM, Beck P, Webster DJT, Roberts CJ (1990). How good are case notes in the audit of radiological investigations?. *Clinical Radiology*.

[11] McKee RF, Scott EM (1987). The value of routine preoperative investigations. *Annals of the Royal College of Surgeons of England*.

[12] (1983). Routine preoperative investigations are expensive and unnecessary. *The Lancet*.

[13] Neragi-Miandoab S, Wayne M, Cioroiu M, Zank LM, Mills C (2010). Preoperative evaluation and a risk assessment in patients undergoing abdominal surgery. *Surgery Today*.

[14] Rucker L, Frye EB, Staten MA (1983). Usefulness of screening chest roentgenograms in preoperative patients. *Journal of the American Medical Association*.

[15] Grier DJ, Watson LJ, Hartnell GG, Wilde P (1993). Are routine chest radiographs prior to angiography of any value?. *Clinical Radiology*.

[16] Stables RH, Trotman-Dickenson B (1994). Prospective assessment of the value of a chest radiograph in the performance of diagnostic cardiac catheterisation in adults. *British Heart Journal*.

[17] Gorry GA, Pauker SG, Schwartz WB (1978). The diagnostic importance of the normal finding. *New England Journal of Medicine*.

[18] Kundel HL (1982). Disease prevalence and radiological decision making. *Investigative Radiology*.

[19] Vecchio TJ (1966). Predictive value of a single diagnostic test in unselected populations. *The New England Journal of Medicine*.

[20] Graat ME, Kröner A, Spronk PE (2007). Elimination of daily routine chest radiographs in a mixed medical-surgical intensive care unit. *Intensive Care Medicine*.

